# Pharmacological Approaches for the Modulation of the Potassium Channel K_V_4.x and KChIPs

**DOI:** 10.3390/ijms22031419

**Published:** 2021-01-31

**Authors:** Pilar Cercós, Diego A. Peraza, Angela de Benito-Bueno, Paula G. Socuéllamos, Abdoul Aziz-Nignan, Dariel Arrechaga-Estévez, Escarle Beato, Emilio Peña-Acevedo, Armando Albert, Juan A. González-Vera, Yoel Rodríguez, Mercedes Martín-Martínez, Carmen Valenzuela, Marta Gutiérrez-Rodríguez

**Affiliations:** 1 Instituto de Química Médica (IQM-CSIC), 28006 Madrid, Spain; pcercos@nebrija.es (P.C.); mmartin@iqm.csic.es (M.M.-M.); 2 Instituto de Investigaciones Biomédicas Alberto Sols (IIBM), CSIC-UAM, 28029 Madrid, Spain; daperaza@iib.uam.es (D.A.P.); adebenito@iib.uam.es (A.d.B.-B.); paulagarcia@iib.uam.es (P.G.S.); 3 Spanish Network for Biomedical Research in Cardiovascular Research (CIBERCV), Instituto de Salud Carlos III, 28029 Madrid, Spain; 4 Department of Natural Sciences, Hostos Community College of CUNY, New York, NY 10451, USA; ANIGN7016@stu.hostos.cuny.edu (A.A.-N.); darielnyc@gmail.com (D.A.-E.); E.Beato2005@gmail.com (E.B.); EPENA4261@stu.hostos.cuny.edu (E.P.-A.); YRODRIGUEZ@hostos.cuny.edu (Y.R.); 5 Instituto de Química Física Rocasolano (IQFR-CSIC), 28006 Madrid, Spain; xalbert@iqfr.csic.es; 6 Departamento de Físicoquímica, Facultad de Farmacia, Universidad de Granada, 18071 Granada, Spain; gonzalezvera@ugr.es

**Keywords:** voltage-gated potassium channels K_V_4, transient outward current, A-type current, potassium channel interacting proteins (KChIPs), protein–protein interactions, K_V_4/KChIPs modulators

## Abstract

Ion channels are macromolecular complexes present in the plasma membrane and intracellular organelles of cells. Dysfunction of ion channels results in a group of disorders named channelopathies, which represent an extraordinary challenge for study and treatment. In this review, we will focus on voltage-gated potassium channels (K_V_), specifically on the K_V_4-family. The activation of these channels generates outward currents operating at subthreshold membrane potentials as recorded from myocardial cells (I_TO_, transient outward current) and from the somata of hippocampal neurons (I_SA_). In the heart, K_V_4 dysfunctions are related to Brugada syndrome, atrial fibrillation, hypertrophy, and heart failure. In hippocampus, K_V_4.x channelopathies are linked to schizophrenia, epilepsy, and Alzheimer’s disease. K_V_4.x channels need to assemble with other accessory subunits (β) to fully reproduce the I_TO_ and I_SA_ currents. β Subunits affect channel gating and/or the traffic to the plasma membrane, and their dysfunctions may influence channel pharmacology. Among K_V_4 regulatory subunits, this review aims to analyze the K_V_4/KChIPs interaction and the effect of small molecule KChIP ligands in the A-type currents generated by the modulation of the K_V_4/KChIP channel complex. Knowledge gained from structural and functional studies using activators or inhibitors of the potassium current mediated by K_V_4/KChIPs will better help understand the underlying mechanism involving K_V_4-mediated-channelopathies, establishing the foundations for drug discovery, and hence their treatments.

## 1. Introduction

Ion channels are macromolecular complexes present in the plasma membrane and intracellular organelles of all cells. They play an important role in the maintenance of cellular integrity, smooth muscle contraction, secretion of hormones, and neurotransmitters, among others. Dysfunction of ion channels results in a group of disorders named channelopathies, which represent an extraordinary challenge for study and treatment [1].

**Figure 1:**
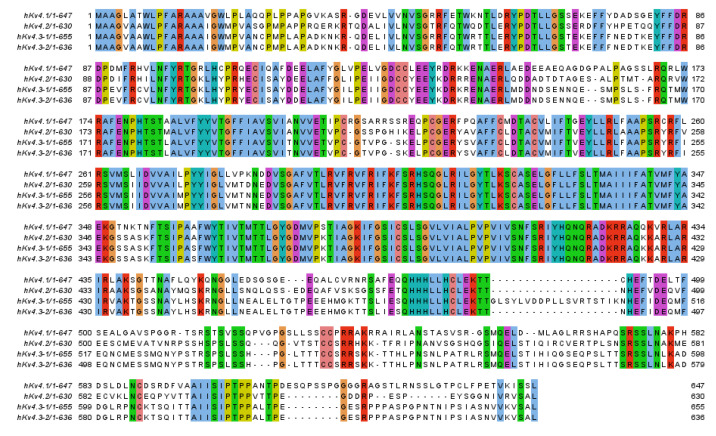
Alignment of human K_V_4. Alignment with Clustal within Jalview. Residues that are identical in the four sequences are boxed [16].

In this review, we will focus on voltage-gated potassium channels (K_V_), specifically on the K_V_4-family or *Shal*-related subfamily. Four members compose this family: K_V_4.1 (*KCND1*), K_V_4.2 (*KCND2*), and two splice variants of K_V_4.3 (*KCND3*), which have an over-all sequence identity of 60% [2,3]. As with other eukaryotic K_V_ channels, K_V_4.x are symmetrically assembled as homo- or heterotetramers of pore-forming subunits (α subunits) around a central pore [4,5]. Each of these α subunits have six transmembrane helices (S1–S6), with its N- and C-terminus pointing to the cytoplasm. At the N-terminus, the principal motif is the so-called T1 domain, which governs the specificity of assembly and is involved in channel tetramerization and its gating. The first four helices (S1–S4) form the voltage-sensing domain, whereas S5, S6, and the P-loop that link them, form the pore domain. There is homogeneity within the K_V_4.x transmembrane region, except in the S6-proximal cytosolic C-terminus, where K_V_4.3 isoform 1 has an additional exon that encodes 19 amino acids (residues 488–506) (Figure 1). Owing to alternative splicing of this additional exon, K_V_4.3 has two variants in human tissues: K_V_4.3L (long form) and K_V_4.3S (short form). Both splice variants are identical except for the additional sequence of 19 amino acids present in the long form K_V_4.3L [6,7].

K_V_4 channels are highly expressed in smooth muscles, heart, and brain. The activation of these channels generates outward currents operating at subthreshold membrane potentials, as recorded from myocardial cells (I_TO_, transient outward current) and from the somata of hippocampal neurons (I_SA_, somato-dendritic subthreshold-activating A-type K^+^ current) [3,8,9]. Among K_V_4 channels, K_V_4.2 and K_V_4.3 are the most thoroughly studied members of the family. Concerning the myocardium, K_V_4.2 and K_V_4.3 present a complementary expression, with K_V_4.2 being more abundant in the ventricle and K_V_4.3 in the atria [8,9]. In the heart, K_V_4 dysfunctions are involved in Brugada syndrome, atrial fibri-llation, hypertrophy, and heart failure [10].

In the brain, the K_V_4 channel expression depends on the neuron type and region, and continuous efforts are made to unravel the organization of these channels in different subcellular compartments and neuron types [11,12]. In hippocampus, K_V_4 channelopathies are linked to schizophrenia, epilepsy, and Alzheimer’s disease [13,14,15]. Hence, the pharmacological modulation of I_TO_ and I_SA_ may have therapeutic value in the treatment of these pathologies.

The α subunits of K_V_4 channels are sufficient for the formation of functional K^+^ channels. However, only the expression of K_V_4 α subunits does not fully reproduce the I_TO_ and I_SA_ currents [17]. K_V_4 need to assemble with other accessory subunits, forming macromolecular complexes with different regulatory (or β) subunits, including KChIPs (potassium channel interacting proteins), DPPs (dipeptidyl-peptidase-like proteins), KCNEs (also termed MinK-related peptides, or MiRPs), KChAPs, and Kvβx subunits [17,18,19,20]. Likewise, these channels are susceptible to be phosphorylated by PKA, PKC, PI-4K-beta, MAPKs, SRC kinases, and so on [21,22,23]. Moreover, it has been described that K_V_4.3 and Na_V_1.5 influence the function of each other [24,25]; the existence of a channelosome formed by K_V_4.3, KChIP3, and Ca_V_3.1 channels has also been described [26,27]. The expression of β subunits varies both among organs and/or among different regions of the same organ [3,12,28,29,30,31,32,33]. Therefore, the function of these channels depends not only on the tissue where they are expressed, but also on the signaling context, involving accessory subunits, kinases, or even other ion channels. These β subunits affect channel gating and/or the traffic to the plasma membrane, and their dysfunctions may affect channel pharmacology [20,34,35].

This review aims to analyze the K_V_4/KChIPs interaction and the effect of small molecule KChIP ligands in the A-type currents generated by the modulation of the K_V_4/KChIP channel complexes. Knowledge gained from structural and functional studies using activators or inhibitors of the potassium current mediated by K_V_4/KChIPs will contribute to understanding the mechanisms involved in the K_V_4-mediated-*c*hannelopathies and to establish the bases of drug discovery to treat them.

## 2. Potassium Channel Interacting Proteins (KChIPs)

**Figure 2:**
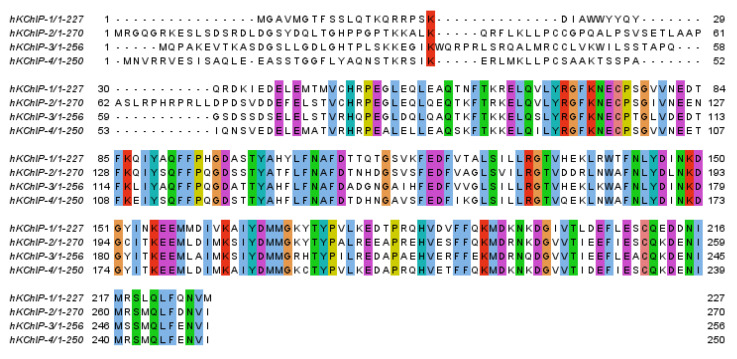
Alignment of human KChIPs. Alignment with ClustalO within Jalview. Residues that are identical in the four sequences are boxed [16].

KChIPs belong to the calcium binding protein superfamily. In mammals, the KChIP subfamily is composed of four members, KChIP1, KChIP2, KChIP3 (also known as DREAM or calsenilin), and KChIP4 (also known as calsenilin-like protein), encoded by four genes (*KCNIP1-4*), all of them with several spliced variants [36,37,38,39]. KChIP1, KChIP3, and KChIP4 are brain-predominant, while KChIP2 is predominantly expressed in both the heart and brain [40]. The four KChIP isoforms have a variable N-terminal region, where the main variations in the sequence as well as a conserved C-terminal domain can be observed, in which four EF-hand-like domains are located (Figure 2) [41]. Apart from KChIP’s activity on the Kv4 modulation, they also show a plethora of functions including the modulation of cardiac and neuronal excitability, presenilin-binding proteins, and calcium-dependent transcriptional mediators [42,43,44].

### 2.1. Three-Dimensional Structures of Free KChIPs

**Figure 3:**
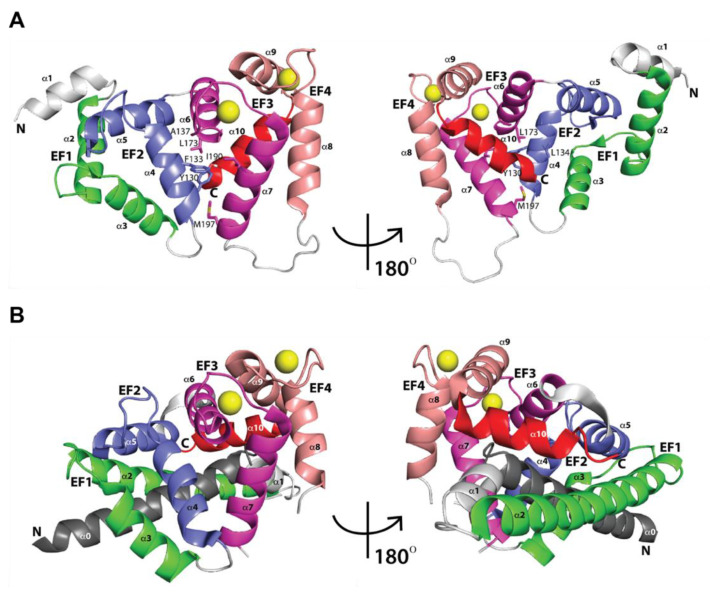
Three-dimensional structures of free KChIPs. The four EF-hand motifs are shown, as representative examples: (**A**) KChIP3 (PDB ID 2JUL) [51] and (**B**) KChIP4a (PDB ID 3DD4) [52]. Different from KChIP1-3, KChIP4a C-terminal α-helix H10 (red) swings outward at ~45° from the hydrophobic pocket. Ca^2+^ ions are shown as yellow spheres.

As of today, few three-dimensional (3D) structures of KChIPs have been determined (PDB IDs 1S1E, 2E6W, 2JUL, 3DD4) using either nuclear magnetic resonance (NMR) or X-ray diffraction techniques. In 2004, one of the first KChIP structures was solved at 2.30 Å resolution by Scannevin et al., corresponding to KChIP1 (PDB ID 1S1E) [45]. Scannevin et al.’s study aimed to better understand the interaction of KChIP1 with K_V_4 subunits. The KChIP1 structure has mainly two regions; the N-terminal one comprising residues 12–95 and the C-terminal one comprising residues 96–192. The N-terminal region houses five α helices (H1 through H5), while the C-terminal houses four α helices (H6 through H9). As has been shown in other calcium binding proteins, KChIP1 exhibits the classical EF-hand, consisting of a helix-turn-helix motif [46,47,48,49]. The pairs of EF-hands are connected by linker loops in a U shape. Specifically, for KChIP1, the first and second EF-hands in the N-terminal region are formed by helices H2 and H3 and helices H4 and H5, respectively. In the C-terminal region of KChIP1, helices H6 and H7 and helices H8 and H9 form the third and fourth EF-hands, respectively (see Figure 3A). H10 is not involved in any EF-hand motif and corresponds to the C-terminal helix and engages in a network of hydrophobic interactions with a crevice formed between Leu127, Thr130, Tyr134, and Ile154 of EF-hand 3 and Phe178, Met182, Phe195, and Cys199 of EF-hand 4 (see Figure 3A). In addition, the X-ray crystal structure of KChIP1 showed only two bound Ca^2+^ ions located in EF-hands 3 and 4. EF-hands 1 and 2 lack the canonical sequence for Ca^2+^ coordination, explaining the lack of Ca^2+^ binding. A few years later, in 2007, the solution NMR structure of KChIP3 EF3-4 (PDB ID 2E6W) became available, consisting of five α helices and a short two-stranded antiparallel β-sheet [50]. EF-hand 3 is formed by α helix H1, β1-strand, and α helix H2, and EF-hand 4 is formed by α helix H3, β2-strand, and α helix H4. Two bound Ca^2+^ ions were also located in EF-hands 3 and 4. H5 is the C-terminal helix. Different from the KChIP1 structure (PDB ID 1S1E), the KChIP3 EF3-4 structure (PDB ID 2E6W) showed a disordered long loop connecting EF-hands 3 and 4, which, due to its flexibility, is thought to function as fingers during calcium signaling or other molecules’ binding and recognition. In 2008, Lusin et al. determined another solution NMR structure of KChIP3 (PDB ID 2JUL) [51]. This NMR KChIP3 structure unveiled a similar overall folding as KChIP1 (PDB ID 1S1E). Within the C-terminal region of KChIP3, there are two domains: one comprising EF-hand 1 (residues 90–119) and EF-hand 2 (residues 128–157); and the second one comprising EF-hand 3 (residues 163–192) and EF-hand 4 (residues 211–240). The two domains’ interface forms a cleft through the interactions between EF-hand 2 (Tyr130, Phe133, Leu134, and Ala137) and EF-hand 3 (Leu173, Ile190, and Met197) (see Figure 3A). Likewise, the two bound Ca^2+^ ions are located in EF-hands 3 and 4. In KChIP3, EF-1 is not functional, as its loop’s sequence is not the canonical one for Ca^2+^ binding, and EF-2 bounds Mg. The C-terminal helix (residues 243–254), positioned after EF-hand 4, engages in a network of intramolecular and hydrophobic interactions with a crevice formed between Leu167, Ala170, Met191, and Leu194 of EF-hand 3 and Phe218, Met222, and Cys239 of EF-hand 4. Later on, in 2009, the X-ray crystal structure of KChIP4a was solved at 3.0 Å resolution by Liang et al. (PDB ID 3DD4) [52]. The KChIP4a structure also revealed four EF-hand motifs: six α-helices (H0–H5) comprising the N-terminal region and five α-helices (H6–H10) comprising the C-terminal region (see Figure 3B). Contrary to other KChIPs, the KChIP4a arrangement of the N-terminal α-helices (H0–H2) causes the α-helix H10 to swing outward at ~45° from the hydrophobic pocket, also presented in other KChIPs (see Figure 3A,B). Overall, KChIPs structures have in common the four EF-hand motifs and the two bound Ca^2+^ ions located in EF-hands 3 and 4, but could differ in the arrangement of α-helix H10 with respect to the hydrophobic pocket.

### 2.2. Three-Dimensional Structures of KChIPs-K_V_4.2/K_V_4.3 Channels

**Figure 4:**
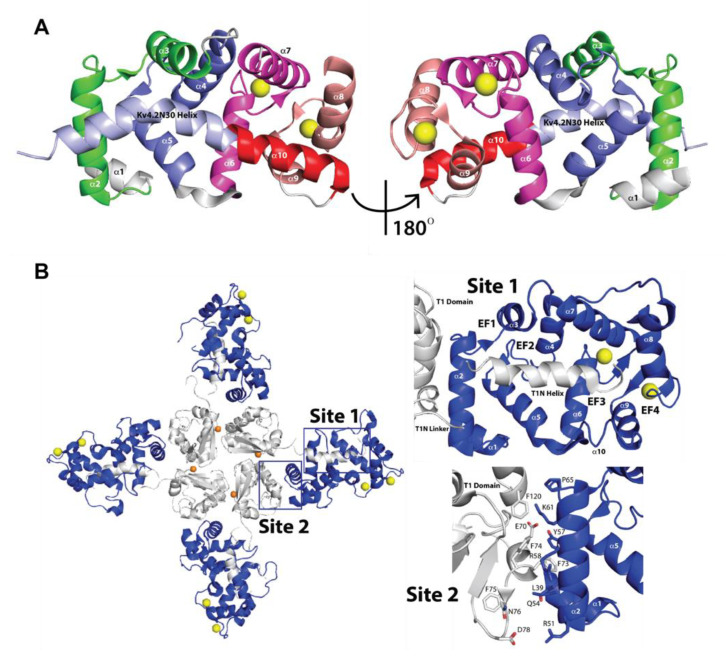
Three-dimensional structures of KChIPs–K_V_4 complexes. (**A**) KChIP1(34–216)–K_V_4.2N30 complex structure (PDB ID 1S6C) showing the four EF-hand motifs (helices H2–H9) and the central hydrophobic pocket occupied by H10 (red) and K_V_4.2N30 (light blue) helices [53]. **(B**) Left panel: Overall structure of the KChIP1–K_V_4.3 T1 complex as seen from the cytosolic side (PDB ID 2I2R) [55]. KChIP1s are shown in dark blue and K_V_4.3 members are shown in white. Ca^2+^ ions are shown as yellow spheres and Zn^2+^ ions are shown in orange. Boxes represent interaction site 1 and interaction site 2. Top right panel: Interaction site 1 showing KChIP1 in dark blue and K_V_4.3 T1N helix, T1N linker, and T1 domain in white. Ca^2+^ ions are shown as yellow spheres. Bottom right panel: Interaction site 2 showing the main interface KChIP1(34–216)-K_V_4.2N30 residues. KChIP1 in dark blue and K_V_4.2N30 residues in white.

To better understand the structural basis of the recognition and modulation of K_V_4 by KChIPs, few X-ray crystal structures of the KChIP1-K_V_4 complex have been determined (PDB IDs 1S6C, 2I2R, 2NZO). In 2004, the first X-ray crystal structure of the core domain of KChIP1(residues 34–216), lacking the N-terminal variable domain, in complex with N-terminal fragment of homoK_V_4.2 (Kv4.2N30, residues 1–30) was solved at 2.0 Å resolution (PDB ID 1S6C) [53]. This complex is a fusion construct made of rat (Rattus norvegicus) KChIP1 (residues 34–216) to the K_V_4.2N30, such that the first residue Met1 of K_V_4.2N30 was linked directly to the last residue Met216 of KChIP1 (residues 34–216). This complex exhibits a clam-shaped dimeric assembly. Four EF-hands form a shell-shaped conformation and create a hydrophobic groove inside the shell. This structure also shows that the first 20 residues of K_V_4.2 form an α-helix (α1), which is kinked around Pro10, a conserved residue among K_V_4 channels. α1 is connected approximately collinearly with the C-terminal α-helix H10 of KChIP1 in head-to-tail fashion, running from the EF-hand 4 end of the shell to the EF-hand 1 end (see Figure 4A). By around the same time the previous structure (PDB ID 1S6C) was reported in 2004, a second structure of KChIP2 in complex with a variant of K_V_4.2 (K_V_4.2*) was reconstructed at 21 Å using negative stain electron microscopy and single particle averaging [54]. Single purified KChIP2-K_V_4.2* channels were visualized in this study. The structure of these channels unveiled that KChIP2 does not interact directly with the membrane domain of the channel to affect function. However, the results indicated that KChIP2 acts through the four internal and four external columns of the K_V_4.2*-KChIP2 channels [54].

Later on, in 2006, a third structure of the human KChIP1-rat K_V_4.3 T1 complex was solved with a 3.35 Å resolution (PDB ID 2I2R) [55]. K_V_4.3 T1 comprises the N-terminal cytoplasmic domain (residues 1–143), while KChIP1 encompasses residues 37–216 containing the core conserved functional of KChIPs. Different from the X-ray crystal structure described above (PDB ID 1S6C), this structure revealed that the assembly between KChIP1 and K_V_4.3 T1 forms a cross-shaped octamer involving the T1 tetramer at the center (see Figure 4B left panel) and individual KChIP1s extending radially (see Figure 4B left panel). In addition, this latter structure (PDB ID 2I2R) displays two main interaction sites. Interaction site 1, with a surface area of ~2100 Å^2^, is located between residues 3 and 21 of a conserved hydrophobic segment on the N-terminal side of T1 (called T1N; Figure 4B left and top right panels) and a large hydrophobic pocket formed by KChIP1. Site 1, different from the complex described above (PDB ID 1S6C), is also formed by a large conformational change in helix H10, which rotates toward the edge of the hydrophobic pocket (see Figure 4B top right panel). T1N is comprised of the following: the T1N helix; an α-helix comprising residues 4–17; and the T1N linker, a loop containing residues 18–39. Both portions contain the essential residues for recognition of K_V_4 by KChIP [36,56]. Conserved T1N residues 7–21 engage in most of the interactions with KChIP1 hydrophobic pocket. K_V_4.3 T1N residues Trp8, Phe11, and Trp19, which are important for KChIP-K_V_4 recognition and current modulation, fit into hydrophobic crevices within the interaction site 1 pocket [53,57]. Trp8 in T1N interacts with Phe60, Val69, Phe74, Ile77, and Phe111 in KChIP1. Phe11 in T1N interacts with Phe98, Phe111, Ala114, Leu115, Leu118, and Trp129 in KChIP1. Trp19 in T1N interacts with Tyr134, Ile150, Ile154, and Tyr155 as well as T1N Met20 in KChIP1. On the other hand, interaction site 2, with a surface area of ~900 Å^2^, encompasses the interaction between the KChIP1 helix H2 and the K_V_4.3 T1 α2-helix and the K_V_4.3 T1 domain “docking loop” (Glu70, Phe73 and Asp78) on the cytoplasmic face of the T1 complex. Phe73 in K_V_4.3 is engaged in an aromatic stacking interaction with Tyr57 in KChIP1. In addition, the side chain of Asp78 in K_V_4.3 forms a salt bridge with Arg51 of KChIP1, while backbone atoms of Glu72 and Phe75 appear to engage in hydrogen bonding with Gln54 in KChIP1. Each KChIP1 interacts with T1N from one K_V_4.3 via interaction site 1, however, KChIP1 also interacts with the adjacent T1 K_V_4.3 monomer via interaction site 2 (see Figure 4B left and bottom right panels). Biochemical and functional studies revealed that site 1 is involved in channel trafficking to plasma membrane, whereas site 2 is involved in channel gating. Afterwards, in 2007, a similar X-ray crystal structure to PDB ID 2I2R of the complex of human KChIP1 and K_V_4.3 N-terminus was solved at a resolution of 3.2 Å (PDB ID 2NZO) [58]. This structure shares similar characteristics to the one described above (PDB ID 2I2R) [55]. The KChIP1-K_V_4.3 complex exhibits the above two interfaces’ interaction, site 1 and site 2. In site 1, the K_V_4.3 N-terminal peptide binds to the KChIP1 deep hydrophobic pocket, replacing helix H10 of KChIP1, which modified the inactivation kinetics of K_V_4. As this binding occurs, a notable conformation change in helix H10 of KChIP1 takes place. Helix H10 swings outward ~40° from the hydrophobic groove. In addition, helices H2 and H8 translate outward by ~2.5 Å and ~1.5 Å, respectively, facilitating the configuration of the peptide binding pocket on the KChIP1 surface. In site 2, KChIP1 interacts with a neighboring K_V_4.3N, different from the one in site 1, via KChIP1 helix H2 and residues 70–78 of K_V_4.3. Phe73, conserved among the K_V_4 members, but not among the other K_V_ channels, fits tightly into a hydrophobic cavity formed by Leu39, Leu42, Leu53, Tyr57, and Phe108 in KChIP1. Additionally, salt bridges seem to be formed between Glu77 and Asp78 of Kv4.3N and Lys50 and Arg51 of KChIP1, respectively. Another conserved residue among the K_V_4 members, Glu70, is engaged via a salt bridge with Lys61 in KChIP1. As Lys61 is conserved among KChIPs, but not in other EF-hand proteins including frequenin and recoverin, which suggests that salt bridges could play important roles in the recognition of K_V_4 by KChIPs (PDB ID 2NZO) [58]. Taken together, the KChIP1-K_V_4.3 structure reveals a clamping mode of the complex, where a single KChIP1monomer sideways clamps two neighboring K_V_4.3 N-termini [55,58]. In addition, the α-helix H10 of KChIPs could remain in the hydrophobic pocket and connect with α1 helix of Kv4.2, as shown in the complex structure of KChIP1 (residues 34–216) to the K_V_4.2N30 (PDB ID 1S6C), in which the first residue Met1 of K_V_4.2N30 was linked directly to the last residue Met216 of KChIP1 (residues 34–216) [53]; alternatively, it could move outward from the hydrophobic pocket, being replaced by α1 helix of K_V_4.3, as shown in the KChIP1-K_V_4.3 structures (PDB IDs 2I2R and 2NZO) [55,58].

## 3. KChIP Ligands

**Figure 5:**
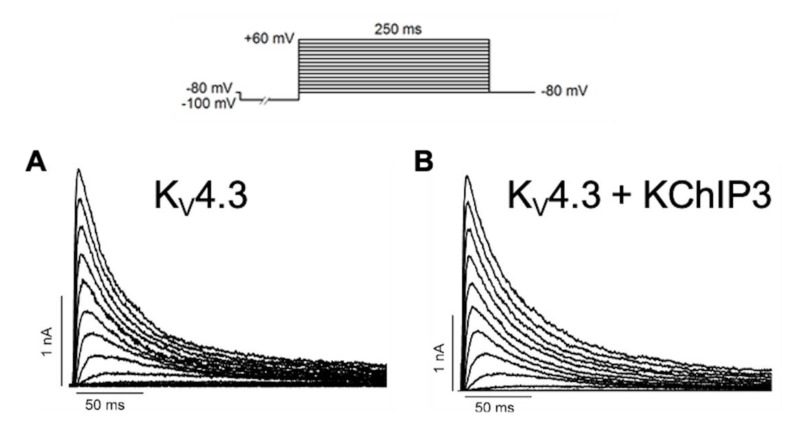
Original K_V_4.3 (**A**) and K_V_4.3+KChIP3 (**B**) records generated after applying the pulse protocol shown in the upper part of the Figure. Note that K_V_4.3+KChIP3 channels exhibit a slower inactivation rate than K_V_4.3 ones.

After the assembly of K_V_4.x channels with KChIP subunits, these regulatory subunits induce an increase in the traffic of K_V_4.x channels to the plasma membrane, a delay in the macroscopic inactivation kinetics, and an acceleration of both the activation and the recovery kinetics from inactivation of K_V_4.x channels (Figure 5) [3,36].

KChIP ligands modify this general trend, and the knowledge gained from the modulation of the K_V_4.x/KChIP complex by small molecules could open new avenues to modulate the potassium outward current through Kv4 channel complexes. At present, eight KChIP ligands have been described as K_V_4.x/KChIP modulators together with structural and/or electrophysiological studies (see Table 1).

**Figure 6:**
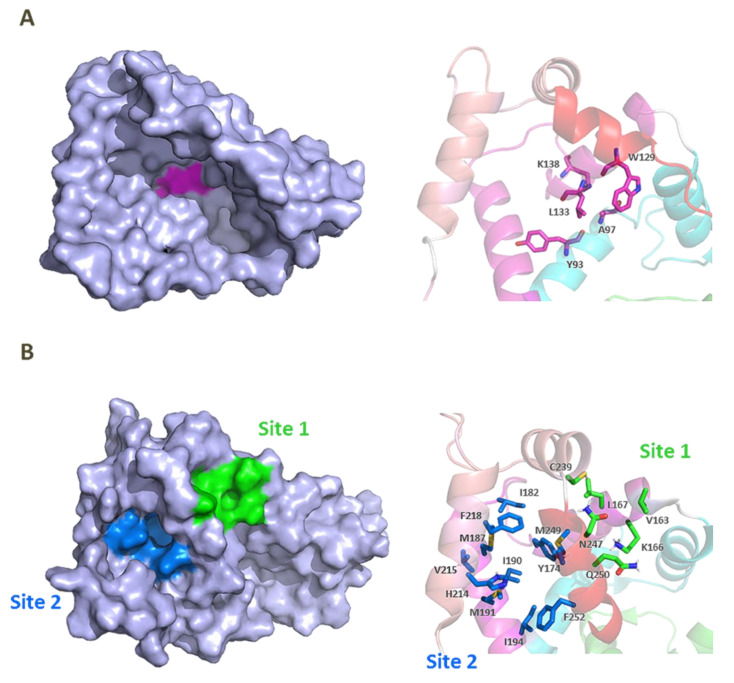
Cartoon and surface representation of (**A**) KChIP1 (PDB ID 2I2R) residues suggested by molecular modeling to be involved in CL-888 binding [59]. (**B**) KChIP3 (PDB ID 2JUL) residues suggested by molecular modeling to participate in 8-anilino-1-naphthalene sulfonate (1,8- ANS) binding [62].

CL-888 is a diaryl-urea compound identified by a yeast two-hybrid assay that binds to KChIP1 and modulates KChIP1 activity on K_V_4.3 channel function. In a surface plasmon resonance assay, CL-888 modifies the apparent affinity of KChIP1 to K_V_4.3-N, but it is not able to dissociate the KChIP1/K_V_4.3-N complex, suggesting a conformational change triggered by the compound binding. Electrophysiological studies with K_V_4.3+KChIP1 channels showed a much greater inhibition of the current generated after the activation of K_V_4.3+KChIP1 channels than that produced by the activation of K_V_4.3 channels [59]. Another study analyzed the effects of CL-888 and repaglinide on K_V_4.3+KChIP3 versus K_V_4.3 expressed in a mammalian cell line [44]. In both studies, it was concluded that CL-888 and repaglinide counteracted KChIPs effects by reducing the maximum peak current and accelerating the decay of the current. Docking studies of CL-888 into the crystal structure of KChIP1 (PDB ID 2S1E) suggested that the binding of this compound is located in a depth relatively hydrophobic cavity of KChIP1, surrounded by Tyr93, Ala97, Trp129, and Leu133 (Figure 6) [59]. To accommodate CL-888, flexibility for Lys138 and Trp129 is necessary (the only Trp in KChIP1). The presence of this Trp residue near the binding site opens the opportunity for fluorescence spectroscopy to analyze the binding of different molecules [59].

Two putative hydrophobic binding sites on KChIP3 surface were identified by fluorescence studies using the fluorescent probes 8-anilino-1-naphthalene sulfonate (1,8-ANS) and 6-anilino 2-naphthalene sulfonate (2,6-ANS), which are highly sensitive to their immediate environment and widely used to study many biological systems (Figure 6) [62]. Additional docking studies using the KChIP3 NMR structure of PDB ID 2JUL provided information about the binding mode of 1,8-ANS and 2,6-ANS toward KChIP3. In these studies, possible binding sites were analyzed and three of them were selected based on the lowest energy and the presence of residues adequate for ANS interaction. Two pockets were located at the C-terminal domain (site 1 and site 2), and one at the EF-1–EF-2 interface. Site 1, between, EF-3 and EF-4, involves Val163, Lys166 (participating in a hydrogen bond with the sulfonate group of ANS), Leu167, Cys239, Asn247, and Gln250. Site 2 is mainly hydrophobic, and it is located near EF-4. In this pocket ANS is surrounded by Tyr174, Ile182, Met187, Ile190, Met191, Ile194, His214, Val215, Phe218, Met249, and Phe252 (Figure 6B). The ability of 1,8-ANS to interact with the C-terminal domain of KChIP3 (161–256) agrees with its binding pocket being located at this domain. Moreover, as the emission spectrum of Trp169 and absorption spectrum of 1,8-ANS overlap, it was determined that the distance between the ANS molecule and Trp169 was 8–16 Å, which was similar in the apo and Ca^2+^ bound KChIP3 and is adequate for the two identified C-terminal sites [62].

In heterologous systems, arachidonic acid modulates the K_V_4.x/KChIPs current [60,61]. In these studies, it was shown that arachidonic acid (AA) decreases the maximum peak amplitude of the currents generated by neuronal, K_V_4.3, and K_V_4.3+KChIP1 channels, with the greater effect being on K_V_4.2/KChIP1. Both analyses showed that AA produced this effect in parallel with an acceleration of the decay of the current. Indeed, one of these studies demonstrated that the inhibitory effects produced by AA are not dependent on the kinetics of the macroscopic speed of the inactivation current. The authors also observed an acceleration of the K_V_4.2 current generated by the activation of K_V_4.2 channels with a deletion in the region of the channel necessary to assembly with KChIP1, whereas this celerity in the macroscopic inactivation is not observed in K_V_4.2 WT channels [61]. Thus, the authors infer that these results are due to the greater degree of inactivation through open channels observed in the presence of KChIP1 rather than in its absence [61,70]. Additionally, it was demonstrated that arachidonic acid directly binds to KChIP3 into site 2 located near EF-4 at the C-terminal domain by 1,8-ANS displacement studies on KChIP3 [62].

NS5806 is another diaryl-urea compound identified as a potassium current activator. NS5806 has been used to study the central role of the transient outward potassium current (I_TO_) on the pathogenesis of the Brugada syndrome (BrS) and to address the molecular composition of cardiac I_TO_ [64,71]. Furthermore, studies with heterologous expressed putative I_TO_ channel subunits indicated that the presence of KChIP2 is mandatory in the modulation of the K_V_ channel produced by NS5806 [63]. Later on, the role of NS5806 on neuronal A-type channel function was also studied, showing that NS5806 is a valuable tool to unravel the subunit composition of native A-type channels in neurons [65].

In 2014, the NS5806 binding to KChIP3 was described [66]. This interaction was first studied by following the fact that the fluorescence emission of the unique tryptophan residue presents in the peptide sequence of KChIP3 upon binding to NS5806. The presence of NS5806 led to a significant diminution in the tryptophan fluorescence emission through a resonance energy transfer process, which correlates well with a dynamic quenching of the excited state of tryptophan. Titration of KChIP3 with increasing amounts of NS5806 showed that this interaction takes place in a Ca^2+^-dependent manner, with a dissociation constant of 2–5 μM in the Ca^2+^-bound form. NS5806/KChIP3 interaction was also characterized by displacement studies in the presence of 1,8-ANS, which confirmed that NS5806 binds to a hydrophobic cavity on KChIP3 near EF-hand 4. Displacement of 1,8-ANS bound to that cavity also allowed to determine the affinities for NS5806 binding to KChIP3, which were very similar to those previously obtained with the quenching data. Fluorescence lifetime measurements showed that the displacement of 1,8-ANS by NS5806 resulted in a significant decrease in the pre-exponential parameter related to 1,8-ANS bound at the solvent restricted hydrophobic cavity. This correlates well with the idea that the KChIP3 binding site is placed in a hydrophobic site and that KChIP3 calcium binding increases availability of the NS5806 to this cavity. The interaction between NS5806 and KChIP3 was also characterized by isothermal calorimetry, indicating that the association process is enthalpy-driven. On the other hand, in the calcium-bound form, F218A showed similar affinity for the K_V_4.3 (2–22) peptide as the wild-type, however, in the presence of the activator, there is a decrease in affinity. These data showed that Tyr174 and Phe218 are important residues for the NS5806 binding, and also have an influence in the interaction with the K_V_4.3-peptide (residues 2–22). Based on these studies, the authors suggested that NS5806 binding led to an increase in H10 flexibility. A 10 ns molecular dynamics simulation showed that, in the presence of NS5806, there is a reorganization of H10 helix and Phe252 to allow the binding of the activator, and that this cavity at the C-terminus can accommodate both the activator and the K_V_4.3 helix [66].

Molecular docking studies using KChIP3 structures (PDB IDs 2JUL and 2E6W) allowed analyzing the binding of NS5806 to KChIP3 [66]. The energetically most favorable binding site was located near EF-3, EF-4, and the H10 helix, with the fluorinated phenyl ring pointing towards the solvent. This binding pocket is similar to the one identified for 1,8-ANS at the C-terminus of KChIP3, which is in agreement with the displacement of 1,8-ANS by NS5806 (Figure 6B) [62]. In the binding pocket, the ligand is surrounded by hydrophobic residues, namely, Tyr174, Ile194, Phe218, and Phe252. NS5806 establishes stacking interactions with Tyr174, Phe218, and Phe252. To ascertain the involvement of Try174, Phe218, and Phe252, site-directed mutagenesis studies were carried out. The replacement of Phe252 by Ala led to a mutant that aggregates, which suggested a role in KChIP3 stability. KChIP3 mutants Y174A or F218A, in the Ca^2+^ bound state, led to two- to threefold decreases in NS5806 affinity, whereas in the apo-state, either there is no change in affinity, as for F218A, or there is a twofold increase, as for Y174A. Y174A mutant also affected the binding affinity of K_V_4.3 (2–22) peptide, leading to a threefold decrease in affinity in the Ca^2+^ bound state, whereas in the presence of NS5806, there is an increase in the affinity of Y174A mutant for this peptide. On the other hand, in the calcium-bond form F218A showed similar affinity for K_V_4.3 (2–22) peptide as wild-type, however, in the presence of the activator, there is a decrease in affinity. These data show that Tyr174 and Phe218 are important residues for NS5806 binding, and also have an influence in the interaction with K_V_4.3-peptide (residues 2–22). Based on these studies, the authors suggested that NS5806 binding led to an increase in H10 flexibility. A 10 ns molecular dynamics simulation showed that, in the presence of NS5806, there is a reorganization of H10 helix and Phe252 to allow the binding of the activator, and that this cavity at the C-terminus can accommodate both the activator and the K_V_4.3 helix [66].

NS3623, a diaryl-urea analogue of NS5806, was first described as a chloride channel inhibitor and later on as a hERG activator [72,73]. Next, in canine preparations, it was described that NS3623 is a dual activator of I_Kr_ and I_TO_ [74]. Although the effects of NS3623 on the K_V_4.3 and HERG channels activation were not mediated by the presence of different β subunits, it seems that KChIP2 and DPP6 modulate the compound’s effects on inactivation kinetics. However, little is known regarding the structural features of the NS3623 binding site on KChIP2 [68].

**Figure 7:**
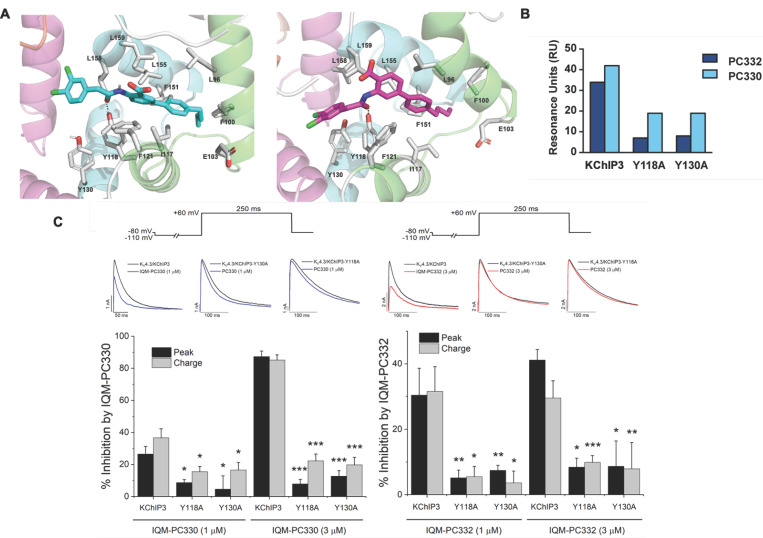
Binding site of IQM-PC330 and IQM-PC332 [69]. (**A**) Cartoon representation of the molecular docking complex of KChIP3 bound to IQM-PC330 (right) and IQM-PC332 (left). (**B**) Direct binding of IQM-PC330 and IQM-PC332 in surface plasmon resonance (SPR) assays to immobilized wtKChIP3 compared with Tyr118Ala and Tyr130Ala KChIP3 mutants. (**C**) Electrophysiological effects of IQM-PC330 and IQM-PC332 on K_V_4.3/KChIP3 wt and K_V_4.3/KChIP3 mutant channels. Data are shown as mean ± SEM. * *p* < 0.05 vs. block produced in K_V_4.3/DREAM wt channels; ** *p* < 0.01 vs. block produced in K_V_4.3/DREAM wt channels; *** *p* < 0.001 vs. block produced in K_V_4.3/DREAM wt channels.

In addition, IQM-PC330 and IQM-PC332 were identified as KChIP3 ligands by a multidisciplinary approach that involved structure-based drug design, organic synthesis, single-site directed mutagenesis, and surface plasmon resonance (SPR) experiments [69]. Starting from CL-888, the urea linker was replaced by an amide linker, and based on computational studies, a virtual compound library was designed, in which an additional substituted-phenyl ring was incorporated to provide further features for a more efficient interaction with KChIP3. Surface plasmon resonance experiments showed improved affinity for KChIP3. To study the binding mode of IQM-PC330 and IQM-PC332 derivatives, molecular docking studies were carried out using homology models of human KChIP3. A search for possible binding pockets was performed, and a site around Tyr118 and Tyr130 was selected for the docking of these two derivatives, which was subsequently refined by molecular dynamics studies (Figure 7A). These studies showed that the n-butyl-biphenyl group is located within a hydrophobic pocket, interacting with Leu96, Phe100, Ile117, Tyr118, Phe121, Phe151, Leu155, Leu159, and the hydrophobic part of Glu103. A greater variability is observed for the binding site of the di-chloro-phenyl moiety between IQM-PC330 and IQM-PC332, although some interactions are shared by them, as the ones with Tyr130 and Leu158. To ascertain the feasibility of this binding pocket, two mutants were prepared, Y118A and Y130A. The binding of both compounds to Y118A and Y130A was evaluated by SPR experiments, showing a decrease in the binding affinity compared with the wild type KChIP3 (Figure 7B).

**Figure 8:**
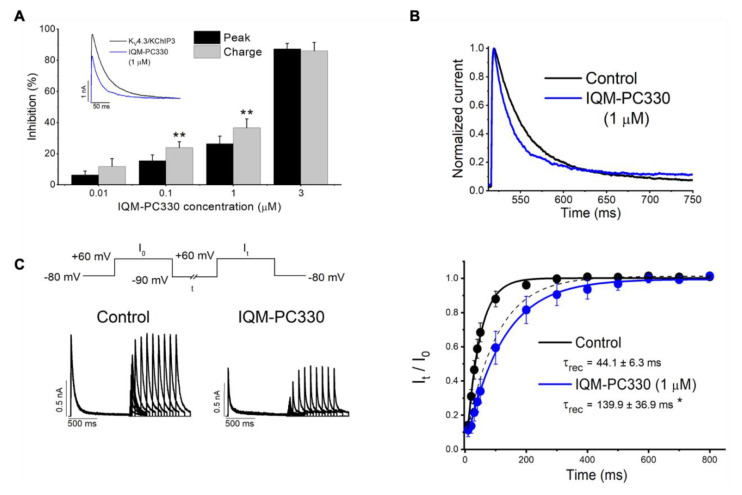
Electrophysiological effects of IQM-PC330 on K_V_4.3 and K_V_4.3/KChIP3 channels [69]. (**A**) Bar chart comparing the inhibition of K_V_4.3/KChIP3 currents produced by IQM-PC330, measured at the maximum peak current and at the charge. Inset shows original current recordings. (**B**) Original normalized current records: control (black line) and with IQM-PC330 (blue line). (**C**) Left panel shows current recordings elicited by the recovery protocol shown in the top. Right panel shows the effects of IQM-PC330 on the recovery process. Dashed lines represent the recovery process of K_V_4.3 current without KChIP3. Note that IQM-PC330 slows the recovery process, reverting KChIP3 effects. Data are shown as mean ± SEM. * *p* < 0.05; ** *p* < 0.01.

In order to evaluate the electrophysiological effects of IQM-PC330 and IQM-PC332 on these potassium channels, experiments on K_V_4.3/KChIP3 channels expressed in a heterologous system were performed (Figure 8). Both compounds inhibited the current with IC_50_ of 1.6 μM and 6.8 μM, respectively. IQM-PC330 accelerated the macroscopic inactivation and, therefore, the inhibition of the charge was greater than that observed when measuring the maximum peak current. On the other hand, IQM-PC332 accelerated this inactivation at low concentrations (between 0.01 and 0.1 μM), producing similar effects to those observed by IQM-PC330. However, at greater concentrations than 1 μM, IQM-PC332 produced opposite effects over the inactivation process and, therefore, its inhibitory effects on the current were greater when measured at the maximum peak current than when measured at the charge. Both compounds slowed the recovery process towards values very close to those recorded on K_V_4.3 in the absence of KChIP3 [69]. In this study, the effect of the Tyr to Ala mutations in the K_V_4.3/KChIP3 channel complex was also analyzed. Expression of KChIP3 Tyr118Ala and KChIP3 Tyr130Ala increased the amplitude of K_V_4.3 current similarly to KChIP3 WT, and accelerated the kinetics of the recovery from inactivation of K_V_4.3 channels. In K_V_4.3/KChIP3-Tyr118Ala and K_V_4.3/KChIP3 Tyr130Ala channels, two concentrations (1 and 3 μM) of each IQM-PC compound were tested. In these experiments, both concentrations produced a negligible inhibition of the current measured both at the charge or at the peak current (Figure 7C,D). Thus, these site-directed mutagenesis results are in agreement with the binding pocket derived from the theoretical studies [69].

**Figure 9:**
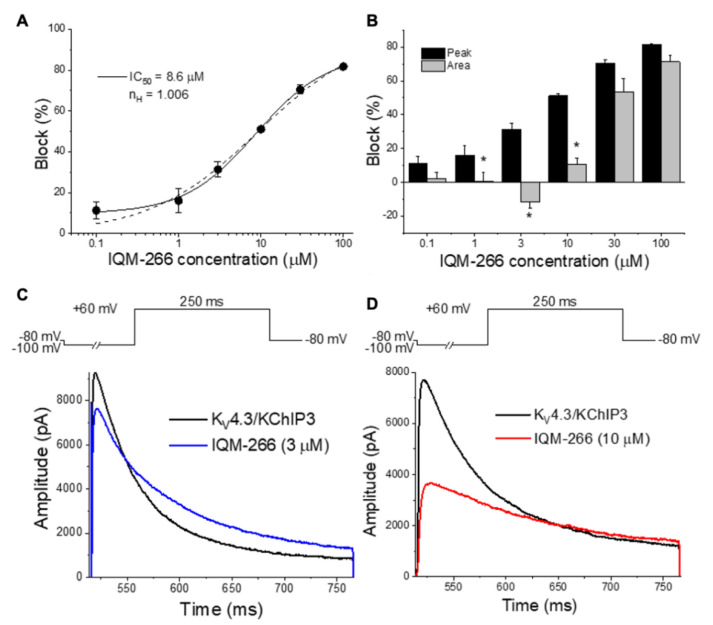
Concentration–dependence of inhibition and/or increase of the current or the charge through K_V_4.3/KChIP3 channels produced by IQM-266 [70]. (**A**) Concentration–response curve of the effects of IQM-266 (continuous line) on K_V_4.3/KChIP3 channels. The dashed line represents the fit of the data to a Hill equation with nH = 1 (*n* = 34). (**B**) Effects on the current induced by IQM-266 in K_V_4.3/KChIP3 channels at the peak current and in the charge (measured as the area of the current after applying 250 ms pulses to +60 mV; *n* = 34). (**C**) Current records of K_V_4.3/KChIP3 to +60 mV in the absence and in the presence of IQM-266 (3 μM). (**D**) Current records of K_V_4.3/KChIP3 to +60 mV in the absence and in the presence of IQM-266 (10 μM). * *p* < 0.05 when comparing the effect of IQM-266 on the peak current and on the charge through K_V_4.3/KChIP3 channels.

IQM-266 was also identified in our Medicinal Chemistry Program oriented to identify KChIP3 ligands [70]. Surface plasmon resonance experiments showed that IQM-266 binds to KChIP3 with a K_D_ = 4.6 ± 0.7 μM. To evaluate the electrophysiological effects of IQM-266, its effects on K_V_4.3/KChIP3 channels were evaluated and compared with those induced on K_V_4.3 channels alone (Figure 9). In these experiments, it was observed that IQM-266 inhibited the K_V_4.3/KChIP3 current in a concentration-, voltage-, and time-dependent-manner. It diminished the maximum peak current at all concentrations tested, but, at a certain range of concentrations, it slowed the inactivation kinetics and IQM-266 produced an increase in the transmembrane charge.

**Figure 10:**
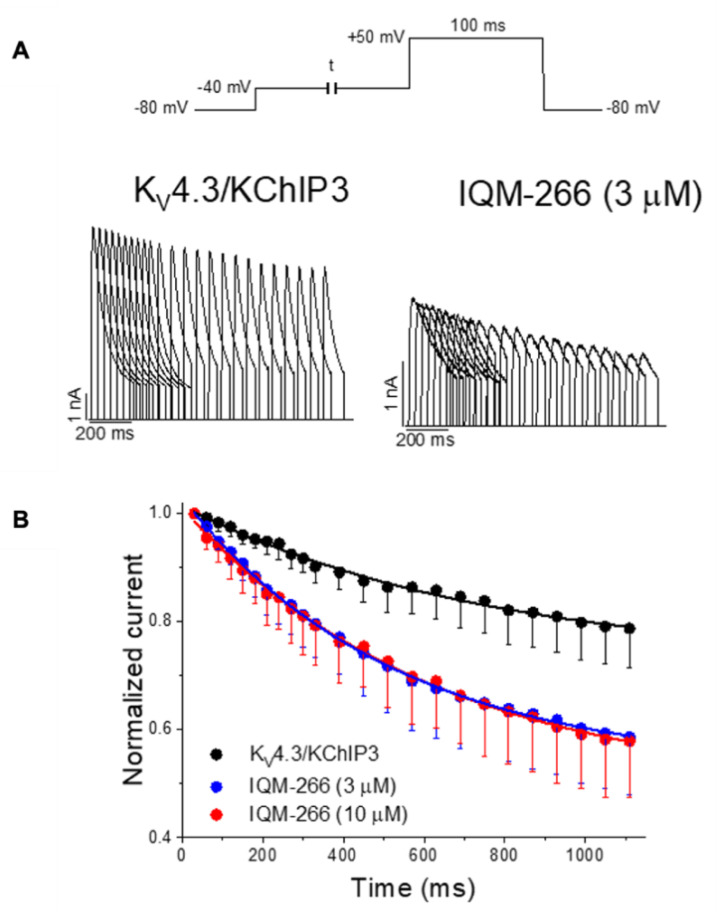
IQM-266 increases the closed-state inactivation of the K_V_4.3/KChIP3 channels [70]. (**A**) Current records generated by K_V_4.3/KChIP3 channels in the absence and in the presence of IQM-266 after applying the pulse protocols shown in the upper part of the figure. (**B**) Time course of closed-state inactivation of K_V_4.3/KChIP3 channels at a membrane potential (−40 mV) at which this current inactivates, but does not conduct. Note how IQM-266 increases the closed-state inactivation.

Similarly to IQM-PC330 and IQM-PC332, IQM-266 slowed the recovery process. Moreover, and as observed with polyunsaturated fatty acids (PUFAs), IQM-266 increased the closed-state inactivation degree, which is consistent with a preferential binding of IQM-266 to a pre-activated closed state of K_V_4.3/KChIP3 channels (Figure 10). The effects of this ligand were also explored in rat dorsal root ganglion neurons. In these cells, IQM-266 inhibited the peak amplitude and slowed the inactivation of I_A_ [70]. Thus, all these results identify IQM-266 as a new chemical tool that might allow a better understanding of KChIP3 physiological function.

## 4. Concluding Remarks

There is a continuous effort to unravel the K_V_4 channelosome in human physiology, its involvement in numerous pathologies, and the search of chemical modulators. The study of K_V_4 channelosome, as well as their regulation by other proteins using novel and selective small molecule ligands, represents pharmacological and molecular challenges. This ongoing effort will assist with the validation of K_V_4 channelosome as therapeutic targets, and the search for better and effective drug candidates for the treatment of neurodegenerative and cardiovascular diseases in which they are involved. Here, we have reviewed K_V_4/KChIPs modulators acting as inhibitors or activators of the A-type currents as pharmacological tools for a better comprehension of the pathophysiological processes in which this channelosome participates. KChIPs modulators have been mainly described in the last decade and used as chemical tools contributing to a better understanding of the K_V_/KChIP interaction.

To study the complex interaction of K_V_4 channelosome and the development of modulators, the interplay of different techniques is central to advancement. In this regard, fluorescent biosensors, a potent tool for probing biomolecules in their native environment and for visualizing dynamic processes, have provided relevant clues regarding the binding site of KChIPs modulators. These tools are well suited for highlighting molecular alterations associated with pathological disorders, thereby offering means of implementation of sensitive and alternative technologies for diagnostic purposes. Therefore, the development of new fluorescent probes with improved photophysical properties and a high signal-to-noise ratio will constitute invaluable tools for the study of the K_V_4 channelosomes with high spatial and temporal resolution.

The size and complexity of protein–protein interfaces make the design of ligand modulators of these interfaces into a challenging task. The knowledge of 3D structures of K_V_ accessory subunits, in particular KChIPs, has provided the identification of regions of the protein structures that can be targeted for the discovery of new molecules that modulate channel activity. This information, together with molecular docking studies, led to the development of new small molecules as K_V_/KChIPs modulators. These promising results will encourage further structural and theoretical studies to gain insights into the molecular determinants of activators and inhibitors of the A-type currents, as well as to design novel compounds as chemical tools to study the protein–protein interaction network of the K_V_4 channelosome. Therefore, they will allow the identification of therapeutically relevant targets and the design of new potential drugs able to modulate them.

The location, expression, or morphological changes of the KChIPs in different tissues, such as the brain [28] or the heart [33], open new opportunities for the treatment of diseases related to these systems. For example, KChIP2 (at the RNA level) is downregulated in several cardiac pathologies (atrial fibrillation, cardiac hypertrophy, or heart failure) [18,40,75]. Thus, the search for new KChIP2 modulators capable of increasing the A-type currents would represent a new family of drugs useful in the treatment of such cardiac pathologies.

Altogether, the knowledge acquired from structural and functional studies using the recently discovered K_V_4/KChIPs modulators and those likely to come soon will help understand the underlying mechanism involving K_V_4-mediated-channelopathies, establishing the foundations for drug discovery, and hence their treatments.

## Figures and Tables

**Table 1:** Known small-molecule K_V_4.x/KChIP modulators.

Ligand	K_V_4.x Channel	KChIPx	Effect	Ref.
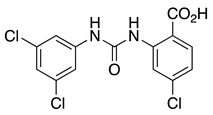 CL-888 (**1**)	K_V_4.2 K_V_4.3	KChIP1 KChIP3	Inhibition	[44,59]
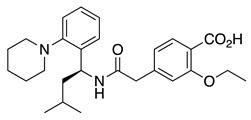 Repaglinide (**2**)	K_V_4.3	KChIP3	Inhibition	[44]
Polyunsaturated fatty acids (PUFAs)	Kv4.3 Kv4.2	KChIP1	Inhibition	[60,61,62]
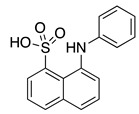 1,8-ANS (**3**)	---	KChIP3	Inhibition	[62]
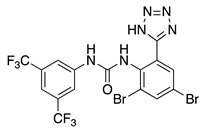 NS5806 (**4**)	K_V_4.3	KChIP2KChIP3	Inhibition/Activation	[63,64,65,66,67]
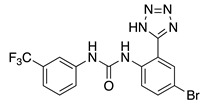 NS3623 (**5**)	K_V_4.3	KChIP2	Inhibition/Activation	[68]
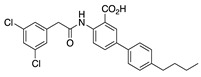 IQM-PC330 (**6**)	K_V_4.3	KChIP3	Inhibition	[69]
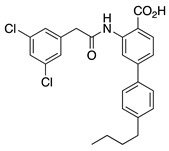 IQM-PC332 (7)	K_V_4.3	KChIP3	Inhibition	[69]
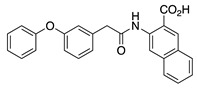 IQM-266 (8)	K_V_4.3	KChIP3	Inhibition/Activation	[70]

## Data Availability

Not applicable.

## Abbreviations

1,8-ANS	8-anilino-1-naphthalene sulfonate
DPPs	Dipeptidyl-peptidase-like proteins
I_SA_	Somato-dendritic subthreshold-activating A-type K+ current
I_TO_	Transient outward current
K_V_	Voltage-gated potassium channels
KChIPs	Potassium channel interacting proteins
MAPKs	Mitogen-activated protein kinase
NMR	Nuclear magnetic resonance
PI4K-beta	Phosphatidylinositol 4-kinase beta
PKA	Protein kinase A
PKC	Protein kinase C
SPR	Surface plasmon resonance
